# Regulation of anti-apoptotic signaling by Kruppel-like factors 4 and 5 mediates lapatinib resistance in breast cancer

**DOI:** 10.1038/cddis.2015.65

**Published:** 2015-03-19

**Authors:** M K Farrugia, S B Sharma, C-C Lin, S L McLaughlin, D B Vanderbilt, A G Ammer, M A Salkeni, P Stoilov, Y M Agazie, C J Creighton, J M Ruppert

**Affiliations:** 1 Department of Biochemistry, West Virginia University Health Sciences Center, Morgantown, WV 26506, USA; 2 Program in Cancer Cell Biology, West Virginia University, Morgantown, WV 26506, USA; 3 The Mary Babb Randolph Cancer Center, West Virginia University, Morgantown, WV 26506, USA; 4 Department of Medicine, West Virginia University, Morgantown, WV 26506, USA; 5 Department of Medicine and Dan L Duncan Cancer Center, Division of Biostatistics, Baylor College of Medicine, Houston, TX 77030, USA

## Abstract

The Kruppel-like transcription factors (KLFs) 4 and 5 (KLF4/5) are coexpressed in mouse embryonic stem cells, where they function redundantly to maintain pluripotency. In mammary carcinoma, KLF4/5 can each impact the malignant phenotype, but potential linkages to drug resistance remain unclear. In primary human breast cancers, we observed a positive correlation between *KLF4*/*5* transcript abundance, particularly in the human epidermal growth factor receptor 2 (HER2)-enriched subtype. Furthermore, KLF4/5 protein was rapidly upregulated in human breast cancer cells following treatment with the HER2/epidermal growth factor receptor inhibitor, lapatinib. In addition, we observed a positive correlation between these factors in the primary tumors of genetically engineered mouse models (GEMMs). In particular, the levels of both factors were enriched in the basal-like tumors of the C3(1) TAg (SV40 large T antigen transgenic mice under control of the C3(1)/prostatein promoter) GEMM. Using tumor cells derived from this model as well as human breast cancer cells, suppression of KLF4 and/or KLF5 sensitized HER2-overexpressing cells to lapatinib. Indicating cooperativity, greater effects were observed when both genes were depleted. KLF4/5-deficient cells had reduced basal mRNA and protein levels of the anti-apoptotic factors myeloid cell leukemia 1 (MCL1) and B-cell lymphoma-extra large (BCL-XL). Moreover, MCL1 was upregulated by lapatinib in a KLF4/5-dependent manner, and enforced expression of MCL1 in KLF4/5-deficient cells restored drug resistance. In addition, combined suppression of KLF4/5 in cultured tumor cells additively inhibited anchorage-independent growth, resistance to anoikis and tumor formation in immunocompromised mice. Consistent with their cooperative role in drug resistance and other malignant properties, *KLF4/5* levels selectively stratified human HER2-enriched breast cancer by distant metastasis-free survival. These results identify KLF4 and KLF5 as cooperating protumorigenic factors and critical participants in resistance to lapatinib, furthering the rationale for combining anti-MCL1/BCL-XL inhibitors with conventional HER2-targeted therapies.

In mouse embryonic stem (ES) cells, pluripotency is maintained by the redundant function of three Kruppel-like transcription factors, KLF2, KLF4 and KLF5.^1^ Furthermore, as determined by chromatin immunoprecipitation combined with high-throughput sequence analysis (ChIP-seq), KLF4 and KLF5 (KLF4/5) have both overlapping and distinct target genes.^2^ Depletion of *Klf4* or *Klf5* in the anterior eye elicits similar developmental phenotypes, whereas in other tissues they exert opposing influences.^3, 4, 5^ For example, KLF4/5 differentially affect the expression of several cell cycle regulatory proteins, such as CCND1, CCNB1 and p21^Waf1/Cip1^.^3^ In adult tissues, KLF4 and/or KLF5 are induced by a variety of stress stimuli and can promote cell survival in diverse contexts.^3, 6, 7, 8, 9^

In breast cancer, KLF4/5 protein levels or mRNA abundance are elevated in aggressive primary tumors.^10, 11, 12, 13^ Consistent with these results, promoter demethylation of *KLF4* or *KLF5* in breast tumors is associated with an unfavorable clinical course.^14^ Individually, both KLF4/5 exert oncogenic functions in experimental models of cancer such as cellular transformation, migration, invasion and xenograft formation.^15, 16, 17, 18, 19^ Although signaling mechanisms remain to be elucidated, KLF4 directly regulates the transcription of microRNA-206 (miR-206) to promote tumor cell survival and tumor initiation in athymic mice (manuscript submitted).^18, 20^ Although independently KLF4/5 have important roles in breast cancer, the relationship between the two genes in this disease remains understudied.

We observed a positive correlation of *KLF4*/*5* expression in the human epidermal growth factor receptor 2 (HER2)-enriched breast cancer subtype. In addition, in these patients the median expression of both *KLF4*/*5* significantly stratified the distant metastasis-free survival (DMFS). Clinically approved HER2-targeted therapies such as lapatinib and trastuzumab (Herceptin) have significantly improved the disease-free survival (DFS) of patients with HER2-amplified breast cancers.^21, 22^ However, eventual resistance to these therapies is observed in the majority of cases, representing a major obstacle to long-term cures.^23, 24, 25, 26^

Several mechanisms of resistance have been described, often involving sustained signaling through dimerization with other receptor tyrosine kinases (RTKs) or activating mutations in downstream effectors, namely RAS pathway components.^27, 28, 29, 30, 31, 32^ Although numerous pathways to resistance have been characterized, it is unclear which of these mechanisms predominate in patients and how they are specifically regulated.

Interestingly, neutralization of apoptotic signaling contributes to anti-HER2 therapeutic failure.^33, 34, 35^ For example, phosphorylation of BAD or overexpression of B-cell lymphoma-extra large (BCL-XL) reduces the efficacy of these drugs. One such drug is lapatinib, a HER2/epidermal growth factor receptor (EGFR) inhibitor that has activity not only in the HER2+ cancers of patients, but also shows efficacy in combination with other agents in basal-like breast cancer models.^36, 37^ We observed that lapatinib treatment of HER2-amplified tumor cells resulted in the rapid induction of KLF4/5. Subsequent experiments demonstrated a novel role for the endogenous KLFs in the regulation of anti-apoptotic factors. As shown by shRNA studies, in the presence of lapatinib KLF4/5 coregulated the expression of myeloid leukemia cell 1 (MCL1) and cooperated to confer lapatinib resistance. Even in the absence of drug treatment, the endogenous KLFs were critical for maintaining basal levels of the anti-apoptotic factors MCL1 and BCL-XL, and collaboratively promoted the malignant phenotype. KLF4/5 were positively correlated with MCL1 in primary breast tumors, and enforced expression of MCL1 was sufficient to rescue the lapatinib sensitivity of KLF4/5-deficient cells. These results identify KLF4/5 as inducible regulators of lapatinib resistance through mediation of anti-apoptotic signaling.

## Results

### *Klf4* and *Klf5* are differentially expressed and positively correlated in genetically engineered mouse models (GEMMs) of breast cancer

To better understand how the expression of these two KLFs are altered during mammary tumorigenesis, we analyzed the levels of *Klf4*/*5* in GEMMs of breast cancer.^38^ As a complement to human tumor analysis, individual GEMMs offer a genetically homogenous background where tumors arise in the context of specific genetic alterations.

**Figure 1:**
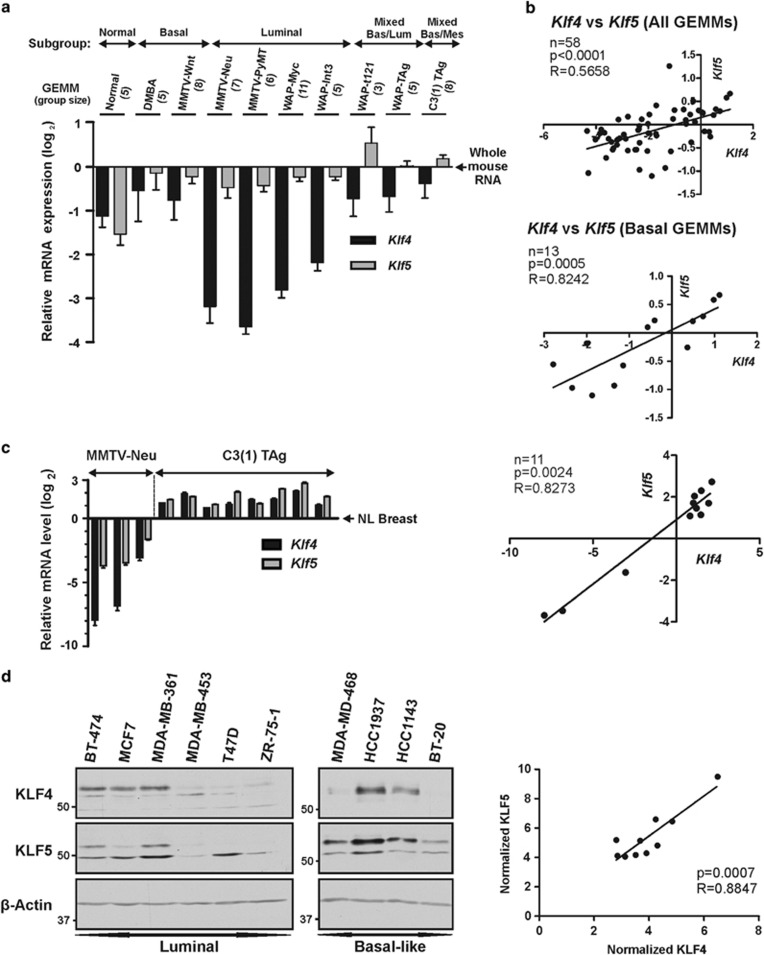
*Klf4* and *Klf5* are differentially expressed and positively correlated in GEMMs of breast cancer. (**a**) Microarray analysis of *Klf4*/*5* levels across GEMMs of breast cancer. Data for 58 mammary tumors from the Gene Expression Omnibus (GSE3165) were organized by GEMM and molecular subtype. Expression values were normalized to whole mouse RNA *(bars,* S.D.). *Klf4* levels in luminal and non-luminal tumors were compared via one-way ANOVA using Dunnet's *post test* (*P*<0.0001). (**b**) Spearman's correlation was performed for the samples in panel **a**. (**c**) qRT-PCR analysis of total RNA isolated from tumors of MMTV-Neu or C3(1) TAg transgenic mice (left panel). Normal mammary tissue from FVB/N mice was analyzed similarly (NL breast, *N*=3). Tumor mean expression is depicted relative to the mean for normal tissue (bars, S.E.). The overall mean tumor expression of *Klf4* and *Klf5* was compared between GEMMs using a two-tailed *t*-test (for each gene, *P*<0.0001). The log_2_ transformed data were assessed by Spearman's correlation (right panel). (**d**) Western blot analysis of KLF4/5 levels in whole-cell lysate of 10 different breast cancer cell lines. KLF expression was determined using ImageJ and normalized to *β*-actin. The expression values were assessed by Spearman's correlation (right panel)

Of 108 tumors in the microarray data set, we analyzed 58 tumor samples across 9 different GEMMs.^38^ We omitted models that had very low abundance of *Klf4*/*5,* including p53-deficient models and models on the BALB/c background. We also omitted samples that did not cluster into an intrinsic subtype (14 tumor samples), and we excluded the mesenchymal subgroup tumors because of heterogeneity in GEMM of origin (five tumors) (Figure 1a). Unlike for *Klf5*, *Klf4* expression as determined by microarray analysis varied substantially across the mouse model tumors, with higher expression in mice transgenic for the coding region of SV40 large T antigen driven by the C3(1)/prostatein promoter (i.e., C3(1) TAg) than in mouse mammary tumor virus promoter (MMTV)-Neu tumors. Collectively, *Klf4* levels were significantly lower in GEMMs that generated predominantly luminal tumors relative to tumors with basal characteristics (*P*<0.0001).

Interestingly, the expression of *Klf4*/*5* were positively correlated in the 58 GEMM tumors (*R*=0.5658, *P*<0.0001; Figure 1b, upper panel). Among the tumor subgroups, the strongest correlation was observed in basal tumors (*R*=0.8242, *P*=0.0005; Figure 1b, lower panel). Significant correlations were also present in the luminal tumors (data not shown). Indicating specificity, *Klf4* or *Klf5* did not correlate or trend toward a correlation with another KLF, *Klf2* (data not shown).^1^

To more accurately quantitate gene expression, we isolated spontaneous tumors from the C3(1) TAg and MMTV-Neu models and measured mRNA levels by quantitative reverse transcription and polymerase chain reaction (qRT-PCR; Figure 1c, left panel). In the C3(1) TAg tumors, *Klf4/5* abundance averaged 2.6- and 3.5-fold higher than normal breast, respectively. In contrast, MMTV-Neu tumors showed markedly reduced levels of both factors. The two models differed in average abundance by 158- and 26-fold for *Klf4*/*5*, respectively (*P*<0.0001). In these tumors, *Klf4/5* mRNA correlated strongly (Figure 1c, right panel; *R*=0.8273, *P*=0.0024). Consistent with animal model data, the protein expression of KLF4/5 in 10 different human breast cancer cell lines was positively correlated (*R*= 0.8847, *P*=0.0007; Figure 1d). For KLF5, the smaller fragment of approximately 48 kDa present in the luminal breast cancer cell lines may be attributed to estrogen-dependent processing.^39^

### Prognostic significance of *KLF4* and *KLF5* in HER2-enriched breast cancer

As KLF4/5 were correlated in breast cancer models, we next examined whether this relationship is informative to patient outcome. We performed Kaplan–Meier analyses of DMFS using a previously described compendium of gene expression profiling data sets representing 1065 cases.^40^ Median expression levels were used to define the groups. We excluded normal breast-like tumors from analysis because these samples often contain high amounts of contaminating normal cells.^41^

**Figure 2:**
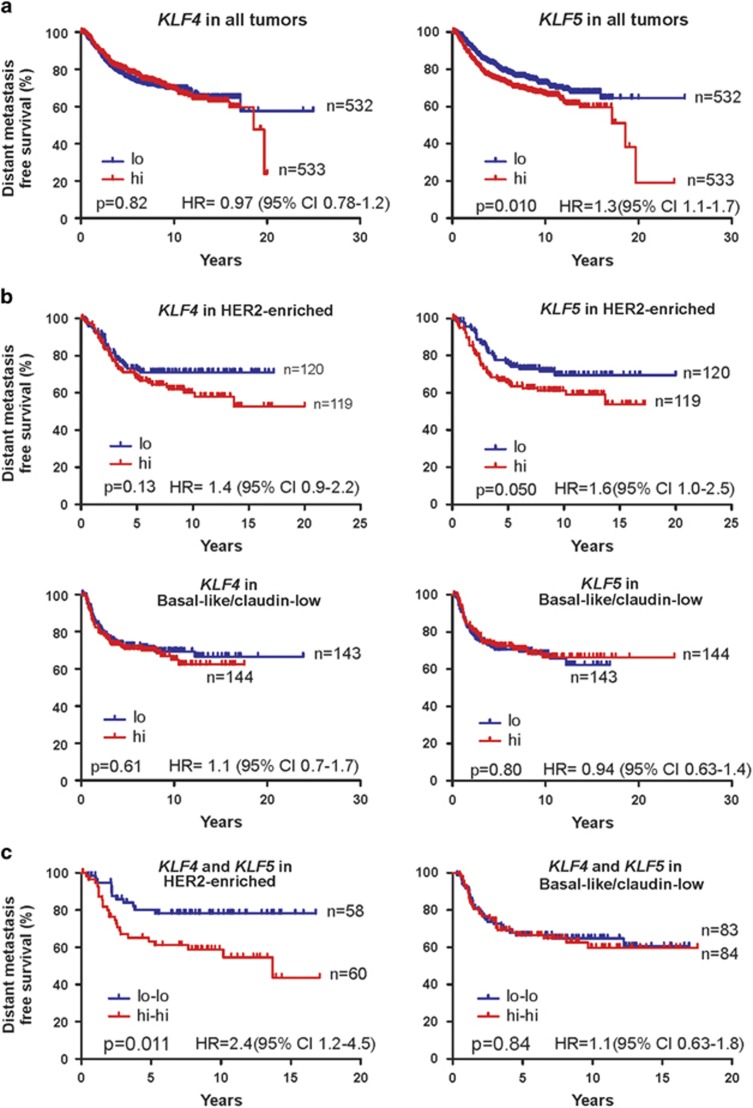
Prognostic significance of *KLF4* and *KLF5* in human breast cancer. Kaplan–Meier analysis utilized a previously described breast cancer microarray database.^40^ Red (hi) and blue (lo) groups were defined using the median gene expression level within the tumors of breast cancer patients. A total of 364 luminal A, 175 luminal B, 239 HER2-enriched and 287 basal-like/claudin-low tumors were analyzed. (**a**) *KLF4*/*5* were analyzed as single variables for all tumors combined. (**b**) *KLF4*/*5* were analyzed as single variables within the basal-like/claudin-low and the HER2-enriched groups, as defined by PAM50 subtyping. (**c**) The outcome of patients harboring tumors with higher expression levels of both KLF4 and KLF5 (red, hi-hi) was compared with the outcome when tumors had lower expression levels of each factor (blue, lo-lo). For all curves, significance was determined using the log-rank (Mantel–Cox) test and *P*<0.05 was considered significant

Across all tumors, *KLF4* levels showed no significant relationship with DMFS (Figure 2a). In contrast, elevated *KLF5* was associated with shortened DMFS, consistent with prior observations (hazard ratio (HR), 1.3; 95% confidence interval (CI), 1.1–1.7; *P*=0.01; Figure 2a).^11^

Interestingly, analysis of DMFS in each intrinsic subtype revealed a trend for increased *KLF5* expression and poor outcome in the HER2-enriched tumors (HR, 1.6; CI, 1.0–2.5, nominal *P*=0.05; Figure 2b). Furthermore, *KLF4* expression showed a similar trend in this context (HR, 1.4; CI, 0.9–2.2, *P*=0.13). No such relationships were observed in a similarly high-risk group, basal-like/claudin-low (Figure 2b), nor in the luminal A or luminal B groups (Supplementary Figure 1). Furthermore, the expression of *KLF4*/*5* together (i.e., < median levels of each gene *versus* ≥ median levels of each gene) considerably stratified DMFS in the HER2-enriched subtype (HR, 2.4; CI, 1.2–4.5, *P*=0.011; Figure 2c). HER2-enriched tumors with elevated *KLF4*/*5* levels had DMFS similar to 43% at 15 years post-diagnosis. This effect was unique to the HER2-enriched subtype, as no other group showed any trend (Figure 2c and Supplementary Figure 1). To assess survival in patients with elevated expression of only one of the two KLFs, we analyzed the four survival curves defined by 2 × 2 contingency using the median scores for *KLF4*/*5* (Supplementary Figure 1). The log-rank test for trend identified a significant trend between KLF4/5 expression and median DMFS (*P*=0.016).

### Endogenous KLF4/5 are induced by lapatinib in breast cancer

**Figure 3:**
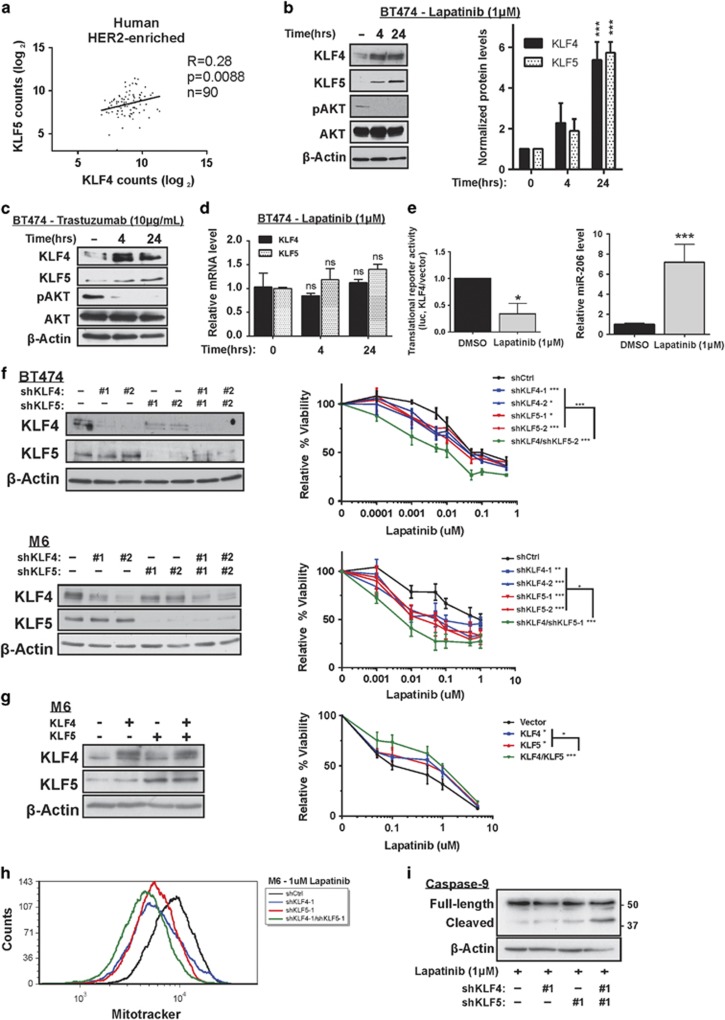
Endogenous KLF4/5 mediate lapatinib resistance in breast cancer models. (**a**) Levels of *KLF4*/*5* in primary human breast tumors were determined by RNAseq (Illumina HiSeq RNAseqV2). Upper quartile normalized data were downloaded from TCGA and assigned a PAM50 subtype. Spearman's correlation was performed on the log_2_ transformed data. (**b**) BT474 cells were treated with DMSO or lapatinib for the indicated interval. Whole-cell lysate was analyzed by western blot. Expression levels from three independent experiments were determined using ImageJ for quantitation, with normalization to *β*-actin (bars, S.D.). (**c**) BT474 cells were treated with trastuzumab or sterile water for the indicated interval and whole-cell lysate was analyzed by western blot. (**d**) *KLF4/5* transcript levels were determined by qRT-PCR following lapatinib exposure. Expression data were normalized using the housekeeping gene *B2M*. (**e**) The pMIR-Report-Luc-KLF4-FL translation reporter contains as an insert within the *FLuc* 3' UTR the full-length *KLF4* transcript, including the *KLF4* protein coding region and the flanking UTRs, as previously described.^18^ Translation efficiency was measured by determining normalized Fluc activity in BT474 cells treated for 24 h with lapatinib or DMSO (left panel). miR-206 levels were determined by qRT-PCR following 24-h lapatinib exposure. Expression data were normalized using U6 snRNA (right panel). (**f**) Cells were treated with the indicated shRNA construct, and the resulting cell populations were treated with lapatinib for 96 h. For each cell population, cell viability relative to the DMSO control was obtained via ATP-based luminescence assay (bars, S.D.). (**g**) Similarly, the lapatinib effect on the relative cell viability of M6 cells expressing ectopic KLF4 and/or KLF5 was determined. Empty vector served as a control. (**h**) To assess MMI, M6 cells were treated with lapatinib for 24 h, stained with 250 nM of Mitotracker dye and analyzed by flow cytometry. (**i**) To assess activity of the intrinsic apoptotic pathway, caspase-9 levels were determined in M6 cells expressing shCtl, shKLF4, shKLF5 or shKLF4/5. Cells were treated with lapatinib for 24 h before preparation of cell extracts. *P<0.05; **P<0.01; ***P<0.001

We next examined the transcript abundance of *KLF4*/*5* as determined by RNAseq analysis of patient breast tumors via The Cancer Genome Atlas (TCGA) Research Network. Among the breast cancer intrinsic subtypes, we observed the expression of the two factors to be most highly correlated in HER2-enriched tumors (Figure 3a and Supplementary Figure 2). Given that *KLF4*/*5* appeared to represent positively correlated prognostic factors in the HER2-enriched breast cancer subtype, we subsequently investigated the interdependence of KLF4/5 expression with exposure to, or resistance to, HER2-targeted therapy.

Interestingly, lapatinib promoted the expression of both the KLF4 and KLF5 proteins (Figure 3b). Trastuzumab treatment yielded similar results (Figure 3c). In these experiments, phosphorylated AKT levels served as a positive control for HER2 inhibition (Figures 3b and c). These effects appeared to be transcriptionally independent, as the respective mRNA levels were not significantly altered (Figure 3d). Nor did enhanced efficiency of protein translation appear to account for upregulation of KLF4. Rather, the translational efficiency of the full-length *KLF4* transcript, as determined using a previously described translation reporter, pMIR-Report-Luc-KLF4-FL, was actually decreased by lapatinib treatment (Figure 3e, left panel).^18^ This decrease is expected when KLF4 transcriptional activity is elevated, attributed to a well-characterized negative feedback signal by which KLF4 induces miR-206 and suppresses its own translation.^18^ Indeed, in lapatinib-treated cells the elevated KLF4 was associated with increased levels of miR-206, which can then directly target the *KLF4* 3' UTR (Figure 3e, right panel).^18, 20^

As neither KLF4 transcription nor its translational efficiency appear to be upregulated by lapatinib, the results are consistent with lapatinib-mediated stabilization of the KLF4 protein. Therefore, lapatinib may function similarly to serum starvation to mediate a prolonged KLF4 half-life.^42^ Several experiments to directly assess half-life were unsuccessful because of the combined toxicity when cells were exposed to both lapatinib and the protein synthesis inhibitor cycloheximide. Nevertheless, the results indicate that lapatinib treatment of HER2-positive breast cancer cells can enhance KLF4 protein expression and its transcriptional activity as indicated by miR-206 levels.

### Endogenous KLF4/5 mediate lapatinib resistance in breast cancer

Based on these results, we hypothesized that KLF4/5 are functionally important in the response to lapatinib. We therefore depleted KLF4/5 in HER2-amplified human BT474 and mouse M6 breast cancer cells, using distinct shRNA hairpins for each of the human and mouse genes (Figure 3f, left panels). M6 cells are a HER2-overexpressing mammary cancer cell line derived from a basal-like GEMM that is enriched for *Klf4/5*, the C3(1) TAg model.^43^ Unlike many basal-like models, M6 cells overexpress both *Egfr* and *Erbb2*, the two RTKs targeted by lapatinib. Compared with the nontargeting control, single knockdown of KLF4 or KLF5 in the BT474 and M6 models significantly sensitized the cells to lapatinib treatment (Figure 3f, right panels). Moreover, coreduction of both KLF4/5 further sensitized the cells to lapatinib, indicating cooperativity. In these experiments, suppression of KLF5 led to a subtle reduction of KLF4, suggesting the possibility of crosstalk between the two factors (Figure 3f, left panels). In agreement with the knockdown studies, ectopic expression of KLF4/5 in M6 enhanced resistance to lapatinib treatment (Figure 3g).

To further characterize their role in drug resistance, we assessed mitochondrial membrane integrity (MMI) in these cells following lapatinib exposure (Figure 3h). Disruption of MMI is a key step of the intrinsic pathway of apoptosis. Following 24 h of lapatinib treatment, the single knockdown cell lines had reduced MMI compared with the controls and suppression of both KLF4/5 produced an additive effect. Supporting an impact of KLF4/5 on the intrinsic pathway of apoptosis, reduction of KLF4/5 enhanced cleaved caspase-9 following exposure to lapatinib, with additive effects when both KLFs were depleted (Figure 3i).

### KLF4/5 cooperate to promote malignant properties

**Figure 4:**
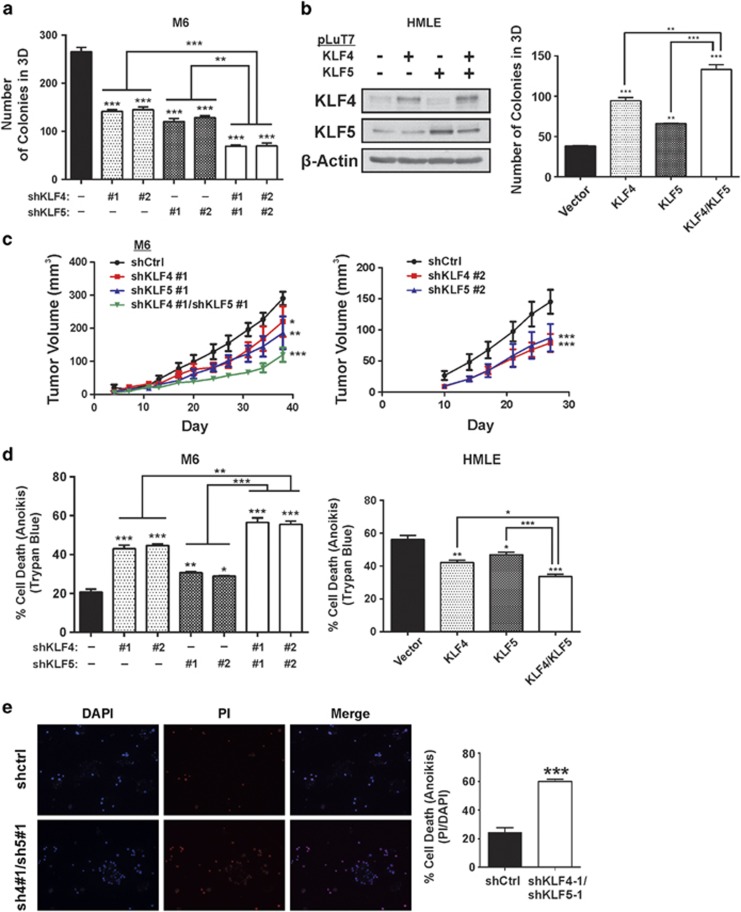
KLF4/5 cooperate to promote malignant properties in M6 cells, a HER-2-overexpressing mammary cancer model. (**a**) Anchorage independence was assessed by incubation of the indicated cell populations in soft agar for 14 days (*N*=3, bars, S.E.). (**b**) Anchorage independence of HMLE cells expressing ectopic KLF4/5 was determined as previously described. Empty vector (–) served as a control so that all cell populations were treated with equal volumes of lentiviral supernatant. (**c**) Cells were injected into the mammary gland of female athymic mice and tumor xenograft volume was monitored over a period of several weeks (left panel, *N*= 5; bars, S.E.). Similar effects on tumor burden were obtained using distinct shRNAs for the suppression of each KLF (right panel, *N*= 5; bars, S.E.). (**d**) Cell death was determined by Trypan blue exclusion following 24 h of matrix deprivation for the indicated cell populations (*N*=3; bars, S.E.). (**e**) As an independent method, cell death because of matrix deprivation in M6 cells was determined by fluorescence microscopic imaging of DAPI/PI-stained cells. Results were quantitated using ImageJ (two-tailed *t*-test; *N*=3; *bars,* S.E.). *P<0.05; **P<0.01; ***P<0.001

We next determined whether KLF4/5 contributed to malignant properties independently of lapatinib exposure. Relative to the control, depletion of either KLF4 or KLF5 in M6 cells significantly impacted anchorage-independent growth, as indicated by reduced colony-forming ability (Figure 4a). Furthermore, there was an additive reduction of colony number following codepletion. We similarly observed cooperativity in a gain-of-function context, as ectopic expression of KLF4/5 enhanced colony-forming ability in immortalized human breast epithelial cells (HMLE, Figure 4b).

Expanding on this observation, we evaluated the ability of these factors to impact tumor formation in athymic mice. Individual reduction of each KLF significantly reduced xenograft growth, with additive effects in the double knockdown cells (Figure 4c, left panel). Comparable results on tumor formation were obtained using an independent shRNA to target each factor (Figure 4c, right panel).

To determine whether these results reflect deficiencies in prosurvival signaling, we examined whether KLF4/5 could cooperatively influence cell death following matrix deprivation (anoikis). Single knockdown of KLF4 or KLF5 sensitized cells to anoikis as determined by Trypan blue exclusion, with cooperative effects following codepletion (Figure 4d, left panel). Conversely, overexpression of these factors in HMLE enhanced anoikis resistance (Figure 4d, right panel). Determination of cell death by an independent method yielded similar results (Figure 4e).

### KLF4/5 depletion is associated with reduced expression of anti-apoptotic B-cell lymphoma 2 (BCL2) family members

As KLF4/5-depleted cells consistently exhibited defects in cell survival, we analyzed molecular effectors of this phenotype. The intrinsic pathway of apoptosis is critical in both lapatinib resistance and anoikis.^44^ We therefore examined the association of KLFs with factors known to participate in apoptotic signaling.

**Figure 5:**
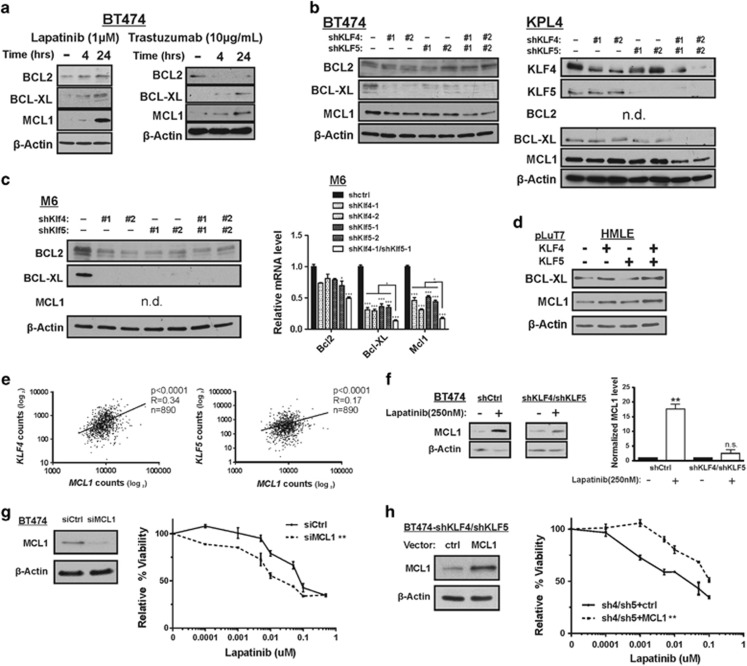
KLF4/5 depletion is associated with reduced expression of anti-apoptotic BCL2 family members. (**a**) BT474 cells were treated with DMSO, 1 *μ*M lapatinib, sterile water or 10 *μ*g/ml trastuzumab for the indicated time intervals. BCL2, BCL-XL and MCL1 levels were determined by western blot analysis. (**b**) Protein expression was analyzed in control or KLF-depleted BT474 and KPL4 cells. (**c**) Protein expression (left panel) and mRNA expression (right panel) was analyzed in the indicated cell populations. (**d**) Protein expression was analyzed in control HMLE cells and in cells expressing ectopic KLF4 and/or KLF5. (**e**) Spearman's correlation between *KLF4*, *KLF5* and *MCL1* levels as determined by RNAseq analysis of 890 human breast tumors. (**f**) The impact of KLF4/5 knockdown on the lapatinib-mediated induction of MCL1 was determined in BT474 cells. Cells were treated with 250 nM lapatinib or DMSO for 24 h. For three independent experiments, the expression levels were quantitated using ImageJ and normalized to *β*-actin (bars, S.D.). (**g**) MCL1 levels were reduced by siRNA and the resulting cell populations were treated with lapatinib for 96 h. For each cell population, cell viability relative to the DMSO control was obtained via ATP-based luminescent assay (paired *t*-test, two tailed; bars, S.D.). (**h**) Similarly, lapatinib resistance was analyzed in KLF4/5 knockdown BT474 cells following rescue with exogenous MCL1 expression vector or empty vector control. *P<0.05; **P<0.01; ***P<0.001

In response to lapatinib or trastuzumab treatment, we observed induction of not only KLF4/5 (Figures 3b and d), but also the anti-apoptotic BCL2 members BCL2, BCL-XL (BCL2L1) and MCL1 (Figure 5a). Although BCL2 was increased in response to lapatinib, BCL2 levels were decreased upon trastuzumab exposure, consistent with previous studies.^45^ Despite the well-documented oncogenic role for BCL2 in hematological malignancies, its expression is correlates with a favorable patient outcome in breast cancer.^46^

Single knockdown of either KLF4 or KLF5 greatly reduced BCL-XL levels in untreated M6 and BT474 cells (Figures 5b and c, left panel). Interestingly, human KPL4 cells required depletion of both KLFs to impact BCL-XL abundance, possibly attributed to the activating phosphatidylinositide 3-kinase (PI3K) mutation present in this HER2-amplified, inflammatory breast cancer cell line (Figure 5b).^47^

In both BT474 and KPL4 cells, a reduction of the MCL1 protein level was evident when KLF4/5 were cosuppressed (Figure 5b). Owing to lack of a suitable antibody, we could not reliably detect murine MCL1 (Figure 5c, left panel). Regardless, in these cells the KLFs cooperated to maintain *Mcl1* and *Bcl-xl* transcript levels, as shown by qRT-PCR (Figure 5c, right panel). Ectopic expression of KLF4/KLF5 in HMLE cells cooperated to increase MCL1 expression, however, BCL-XL levels responded primarily to KLF4 (Figure 5d).

Although the KLFs impacted BCL2 levels in M6 cells, this regulation was not apparent in BT474 cells (Figures 5b and c). qRT-PCR transcript analysis of other BCL2 family members including *Bad*, *Bax* and *Bid* revealed no significant effects by modulation of the two KLFs (data not shown).

To extend these *in vitro* studies, we assessed the copresence of *KLF4/5* expression with *MCL1, BCL-XL* and/or *BCL2* in human tumors (Table 1).^48, 49^ Using a ±1.5 *z*-score range to define high and low expression groups, we evaluated the mutual exclusivity/inclusivity in 958 human breast tumors that were analyzed by microarray. Both *KLF4*/*5* cooccurred with *MCL1*. In addition, *KLF4* and *BCL-XL* expression levels were mutually inclusive, as were *KLF5* and *BCL2*. In agreement with our previous observations (Figure 3a), *KLF4*/*5* expression cooccurred in these tumors.

To more directly test for a correlation in patient samples, we utilized the RNAseq database generated by TCGA. Across 890 breast tumors, the transcript abundance for *KLF4*, *KLF5* and *MCL1* was positively correlated (Figure 5e). Using this data set, we observed no significant positive correlations between KLFs and the other anti-apoptotic genes, *BCL-XL* and *BCL2* (data not shown). Despite these results, extensive ChIP studies in BT474 cells that analyzed 5-kbp upstream of the transcriptional start site, the body of the gene and 3-kbp downstream, failed to identify any KLF4/5 association with the mouse or human *MCL1* loci (see Discussion section).

We next examined whether KLF4/5 expression was required for the induction of anti-apoptotic molecules by lapatinib. We focused on MCL1, as this molecule displayed a robust induction following HER2 inhibition and exhibited consistent relationships with the KLFs in human tumors (Table 1,Figures 5a and e). To ensure a suitable number of viable, lapatinib-sensitive KLF-depleted cells for analysis we used a reduced lapatinib concentration of 250 nM. Although individual reduction of KLF4 or KLF5 did not substantially impact the lapatinib-mediated induction of MCL1 (data not shown), cosuppression of both KLFs blunted this response (Figure 5f).

To validate the importance of MCL1 in lapatinib resistance, we reduced MCL1 levels in BT474 cells using siRNA smart pool (Figure 5g). Compared with the nontargeting control, siMCL1 cells demonstrated increased sensitivity to lapatinib treatment. Similarly, a small molecule MCL1 inhibitor, UMI-77, yielded comparable results to the siRNA studies (data not shown). Conversely, MCL1 overexpression in KLF4/5 knockdown BT474 cells was sufficient to restore lapatinib resistance (Figure 5h).

## Discussion

Targeted therapies have significantly improved the DFS of breast cancer patients, including patients with ER+ or HER2+ breast cancers, and these therapies hold promise for triple-negative breast cancer (TNBC), particularly when used in combination.^36, 37^ For HER2+ breast cancer, clinically approved therapies include the monoclonal antibodies trastuzumab and pertuzumab and, for third- or fourth-line therapy of metastatic disease, the small molecule lapatinib.

For intrinsically aggressive breast cancers such as HER2-enriched tumors, therapeutic resistance is common, as evidenced by the progression of unresponsive tumors.^23, 24, 25, 26^ Similar to lapatinib and trastuzumab, it is likely that resistance mechanisms will impact the newer classes of HER2/EGFR inhibitors, such as neratinib.^50^ Therefore, a better understanding of the molecular drivers of resistance could enable the identification of effective drug combinations to improve clinical efficacy.

Many studies support shared resistance mechanisms between lapatinib and trastuzumab.^27, 28, 29, 30, 31, 32, 35^ These include the sustained activation of downstream effectors of HER2 including PI3K, mTOR and mitogen-activated protein kinase (MAPK). Through its regulation by these pathways or by other signaling entities, the intrinsic cell death pathway confers resistance to anti-HER2 treatments.^33, 34, 35, 45^ Thus, the therapeutic effect of anti-HER2 treatments can likely be modulated using small molecule inhibitors or genetic methods to perturb the balance between pro-apoptotic and anti-apoptotic signaling. For example, a constitutively active pro-apoptotic Bik enhances lapatinib-mediated apoptosis.^33^ Similarly, knockdown or overexpression of either MCL1, BCL2 or BCL-XL significantly alters the efficacy of lapatinib in HER2-amplified cells.^33, 35^

In our study, the KLFs mediated lapatinib resistance in a cooperative manner, a result obtained in HER2-amplified BT474 cells and in M6 cells, a HER2-overexpressing cell line.^38, 51^ Likely contributing to this effect, the two KLFs independently impacted basal levels of the anti-apoptotic factors MCL1 and BCL-XL in drug-naive cells. Furthermore, in lapatinib-treated BT474 cells, we were surprised to observe upregulation of BCL-XL and MCL1 upon HER2 inhibition. The induction of MCL1 required the activity of KLF4/5, as indicated by analysis of the KLF4/5-deficient cells. We are currently assessing other tumor models for KLF4/5-dependent upregulation of anti-apoptotic factors in response to small molecule inhibitors.

There are only a few established links between the KLFs, anti-apoptotic BCL2 family proteins and HER2.^52, 53, 54^ In this study, we observed coexpression of KLF4/5 and MCL1 in human breast tumors and breast cancer models. Also, in mouse ES cells the *Mcl1* locus was significantly enriched via ChIP-seq using antibodies to either KLF4 or KLF5.^2, 55^ Despite these results, and the clear dependence of MCL1 on KLF4/5 expression (Figures 5b and c), the *MCL1* locus was not enriched in KLF4/5 ChIP assays, in which *MIR206* served as a positive control (data not shown).^20^ These results suggest an indirect relationship, although the possibility of direct regulation through a distal binding site cannot be excluded.

We found that *KLF4*/*5* mRNA levels were correlated most strongly in the HER2-enriched tumors, and these factors were selectively prognostic within this subtype. Previous studies have linked adverse clinical outcome to increased nuclear localization of KLF4, to elevated *KLF5* mRNA and to promoter demethylation of *KLF4* or *KLF5*.^10, 11, 12, 13, 14^ In our study, we observed shortened DMFS for patients with HER2-enriched tumors containing elevated levels of both *KLF4* and *KLF5*, implicating these two KLFs as novel prognostic factors (Figure 2d).

In any single breast tumor subtype or in all tumors combined, *KLF4* on its own had little effect (Figure 2b). In contrast to these results, a previous study associated elevated *KLF4* transcript levels with prolonged DFS.^56^ As overall survival, DFS and DMFS correlate strongly in breast cancer, the utilization of these different endpoints seems unlikely to account for the distinct results.^57, 58, 59^ Instead, the discrepancy may be attributed to the analysis of different patient populations, to differences in sample size or to methodological differences in sample processing.

This study identifies a novel drug resistance program composed of KLF4/5, with likely origins in the stress response signaling of KLFs in normal cells. In response to deficient RTK signaling, KLF4/5 can coordinate a prosurvival response that includes BCL2 family proteins, miR-206 and likely other factors. Future studies will examine the predictive utility of KLF4/5 for guiding patient therapy, for example, in patients with HER2+ tumors. Taken together, the identification of KLF4/5 as intermediaries between HER2 and the BCL2 family members significantly strengthens the rationale for combined therapeutic inhibition of HER2 and BCL-XL/MCL1 to combat drug resistance.

## Materials and Methods

### Cell lines and tissue culture

M6 cells were obtained from Jeffrey E Green (National Cancer Institute, Bethesda, MD, USA), KPL4 cells were from Afsaneh Keyhani (MD Anderson, Houston, TX, USA) and HMLE cells were from Robert A Weinberg (Whitehead Institute, Cambridge, MA, USA). Cells were cultured as previously described.^51, 60, 61^ T47D, BT-20, ZR-75-1, MCF7, HCC1937, BT474 and HCC1143 cell lines were purchased from ATCC (Manassas, VA, USA) and cultured in RPMI 1640. MDA-MB-453 cells were cultured in DMEM, whereas MDA-MB-361 and MDA-MB-468 were cultured in DMEM/Ham's F12 50 : 50. All were supplemented with 10% (v/v) FBS (Hyclone, GE Healthcare Life Sciences, Logan, UT, USA), penicillin and streptomycin. Lapatinib (Selleck, Boston, MA, USA) was dissolved in DMSO and used at the indicated concentrations. Trastuzumab (Herceptin) was obtained from the Mary Babb Randolph Cancer Center (Morgantown, WV, USA).

### Transfections and retroviral transduction

The control shRNA vector (nonsilencing-GIPZ lentiviral shRNA control (shCtrl); RHS4346) and the shRNA vectors for murine Klf4 (V3LMM_459916, V3LMM_524009), murine Klf5 (V3LMM_489119, V2LMM_73715) and human KLF5 (V2LHS_150118 and V2LHS_150120) were purchased from Open Biosystems (Pittsburgh, PA, USA). shRNAs targeting human KLF4 have been previously described.^20^

siMCL1 ON-TARGETplus smart pool was purchased from Dharmacon (Lafayette, CO, USA) and transfected using Lipofectamine RNAiMAX Reagent, Life Technologies (Grand Island, NY, USA), as per the manufacturer's recommendations.

Luciferase-based reporter assay of translational efficiency was performed as previously reported, using pMIR-Report-Luc-KLF4-FL and with normalization to pRL-TK.^18^

KLF4/5 were expressed in human cells using the lentiviral vector, pLuT7. KLF4 was ectopically expressed in M6 cells using pBabe-puro-KLF4.^18^ pBabe-puro MCL1 was a gift from Roger Davis (Addgene, Cambridge, MA, USA; plasmid # 25371).^62^ Vectors were packaged into viral particles as previously described.^18^ Cells were infected with viral supernatant supplemented with 10 *μ*g/ml polybrene, centrifuged at 2500 r.p.m. for 1.5 h at 30 °C and selected in puromycin (1.0 *μ*g/ml).

### Immunoblot analysis

Cell lysis, gel electrophoresis, transfer and immunoblot analysis was performed as described.^18^ Primary antibodies were KLF4 (H180, Santa Cruz, Dallas, TX, USA), KLF5 (Millipore, Billerica, MA, USA), MCL1 (S19, Santa Cruz), BCL-XL (L19, Santa Cruz), BCL2 (clone 7/Bcl-2, BD Biosciences, San Jose, CA, USA), phospho-AKT(Ser473, Cell Signaling, Danvers, MA, USA), AKT (Cell Signaling), Caspase-9 (Cell Signaling, 9502) and *β-a*ctin (C-4, Sigma, St. Louis, MO, USA).

### Animal studies

C3(1) TAg mice and MMTV-Neu mice (202Mul) were obtained from Jax Labs (Bar Harbor, ME, USA). Female athymic nude mice (Charles River, Frederick, MD, USA) were obtained at 6–8 weeks of age. In all, 2.0 × 10^6^ cells were injected into the fourth mammary fat pad in 100 *μ*l of DMEM. Caliper measurements were performed twice per week to measure tumor volume. Tumor volume was calculated according to *π*(L1 × L2^2^)/6 (L1, long axis; L2, short axis). All animal procedures were performed under an approved ACUC protocol.

### Quantitative, real-time, reverse transcription and PCR analysis of mRNA

Mouse breast tumors or normal mammary glands were harvested from killed mice and snap frozen. Tissue was mechanically dissociated using glass beads and total RNA was isolated as previously described.^63^ For cell lines, total RNA was extracted using the RNeasy minikit (Qiagen, Valencia, CA). qRT-PCR was performed as previously described.^18^ Primers used for qRT-PCR can be found in Supplementary Table 1. Of the housekeeping genes analyzed using RNA from normal mouse mammary gland and tumors, including *Rplp0*, *Gapdh* and *B2m*, the *Rplp0* levels showed the best correlation with total RNA quantity. Gene expression assays were therefore normalized using this transcript.^64^

### Gene expression analysis of human breast tumors

Data generated using the UNC Illumina HiSeq RNAseqV2 platform were downloaded from TCGA (http://apps.nhlbi.nih.gov/grasp/, 11 March 2013). Statistical programming software R (version 3.0.1) was used to assemble and process the data. Molecular subtyping was accomplished using the Bioconductor 2.12 genefu R package.^65^ At the time of this study, patient follow-up data for TCGA samples were not sufficiently mature for survival analysis. For survival analyses, the compendium of gene expression array data sets of breast cancer was previously compiled, with subtypes assigned as described elsewhere.^40, 66^ Probes used for *KLF4/5* were 221841_s_at and 209211_at, respectively.

### Luminescence based cell viability assay

In all, 2 × 10^3^ cells per well (BT474) or 5 × 10^2^ cells per well (M6) were transferred to 96-well plates and cultured for 96 h in the indicated concentrations of lapatinib. Five replicates were used for each concentration of drug. Fresh media and lapatinib was added to the cells after 48 h. Viable cell number was determined via the ATPlite Luminescence ATP Detection Assay System (PerkinElmer, Waltham, MA, USA). Relative percent viability was determined by normalizing each condition to DMSO treatment only.

### Anoikis sensitivity assays

The anoikis procedure and quantitation of cell death by Trypan blue exclusion was previously described.^20^ Alternatively, cell death was determined using fluorescent microscopic analysis (Zeiss Axio Imager Z2, Oberkochen, Germany) of cells stained with propidium iodide (PI) and 4',6-diamidino-2-phenylindole (DAPI) to identify dead cells and total cell number, respectively.^67^ Quantitation of PI/DAPI-stained cells was performed using Image J (National Institutes of Health, Bethesda, MD, USA).

### Soft agar colony formation

SeaPlaque agarose (Lonza, Anaheim, CA, USA) was dissolved in 1X PBS and autoclaved. The agar layers contained complete growth media and consisted of 1.0 ml 0.5% (w/v) agar underlay, 2.0 ml of 0.5% agar cell layer containing 1.5 × 10^3^ cells/ml and 1 ml of 0.3% agar overlay per well of a six-well plate. In all, 250 *μ*l of growth media was added onto the top layer. The plates were wrapped in parafilm and incubated at 37 °C for 14 days, with an additional 250 *μ*l of complete growth media added after 7 days. Colonies were visualized with a Perfection vV700 Photo scanner (Epson, Long Beach, CA, USA) and colonies >200 *μ*m were counted.

### Flow cytometry

Following 24- h treatment with 1 *μ*M lapatinib, 5.0 × 10^5^ M6 cells were washed twice with PBS, resuspended in 300 *μ*l PBS containing 250 nM Mitotracker Deep Red dye (Invitrogen/GE Healthcare, Logan, UT, USA), and incubated at room temperature for 30 min. Stained cells were centrifuged at low speed and then resuspended in PBS and analyzed in a BD FACS Calibur flow cytometer (BD Biosciences) using BD CellQuest Pro software (BD Biosciences). Plots were generated using FCS Express 4 (*De novo* software, Glendale, CA, USA).

### Statistical analysis

Statistical analyses were performed in GraphPad Prism 6 (GraphPad Software, San Diego, CA, USA). RNA samples were independently analyzed by qRT-PCR three times in duplicate manner (column, mean; bars, S.E.). Correlations were obtained using Spearman's correlation. Xenograft and drug sensitivity assays were analyzed by repeated measures one-way analysis of variance (ANOVA) followed by Tukey's multiple comparison *post test*. Other assays including soft agar growth and anoikis were assessed by one-way ANOVA followed by Tukey's *post test*. Differences were considered significant when two-sided analysis yielded *P* < 0.05.

## Figures and Tables

**Table 1:** The expression levels of KLF4/5 in breast cancer are mutually inclusive with the anti-apoptotic BCL2 family members

*Gene*	*BCL-XL*	*BCL2*	*MCL1*	*KLF5*	*KLF4*
**BCL-XL**	—-	1.22E-1	1.59E-1	1.78E-1	**1.00E-2**^a^
**BCL2**		—-	**6.98E-4**^a^	**2.30E-5**^a^	2.52E-1
**MCL1**			—-	**1.28E-2**^a^	**9.00E-6**^a^
**KLF5**				—-	**<1.00E-6**^a^
**KLF4**					—-

Using cBioPortal, the mutual inclusivity/exclusivity of KLF4/5 and anti-apoptotic BCL2 family member expression was assessed in 958 human breast tumors using a ±1.5 *z*-score range. Transcript abundance was determined by microarray. *P*-values were obtained by Fisher's exact *T*-test (bold indicates significance)

a Denotes an odds ratio (OR) range of 2–10. For this analysis, OR >2.0 signifies cooccurrence of expression within the tumor subsets as defined by the *z*-score

## Abbreviations

BCL2: B-cell lymphoma 2
BCL-XL: B-cell lymphoma-extra large
C3(1) Tag: SV40 large T antigen transgenic mice under control of the C3(1)/prostatein promoter
ChIP-seq: chromatin immunoprecipitation combined with high-throughput sequence
CI: confidence interval
DAPI: 4',6-diamidino-2-phenylindole
DFS: disease-free survival
DMFS: distant metastasis-free survival
ES cells: embryonic stem cells
EGFR: epidermal growth factor receptor
GEMM: genetically engineered mouse model
HER2: human epidermal growth factor receptor 2
KLF: Kruppel-like factor
KLF4/5: KLF4 and KLF5
MAPK: mitogen-activated protein kinase
miR-206: microRNA-206
MMI: mitochondrial membrane integrity
MMTV: mouse mammary tumor virus promoter
MCL1: myeloid cell leukemia 1
PI3K: phosphatidylinositide 3-kinase
qRT-PCR: quantitative reverse transcription and polymerase chain reaction
RTK: receptor tyrosine kinase
TCGA: The Cancer Genome Atlas
TNBC: triple-negative breast cancer
