# Supplementary material for: Regulation of anti-apoptotic signaling by Kruppel-like factors 4 and 5 mediates lapatinib resistance in breast cancer
Source: Cell Death Dis. 2015 Mar 19;6(3):e1699–. doi: 10.1038/cddis.2015.65 (PMC4385942; doi:10.1038/cddis.2015.65)
Supplement: Supplementary Figure 1 [file cddis201565x1.pdf]

A

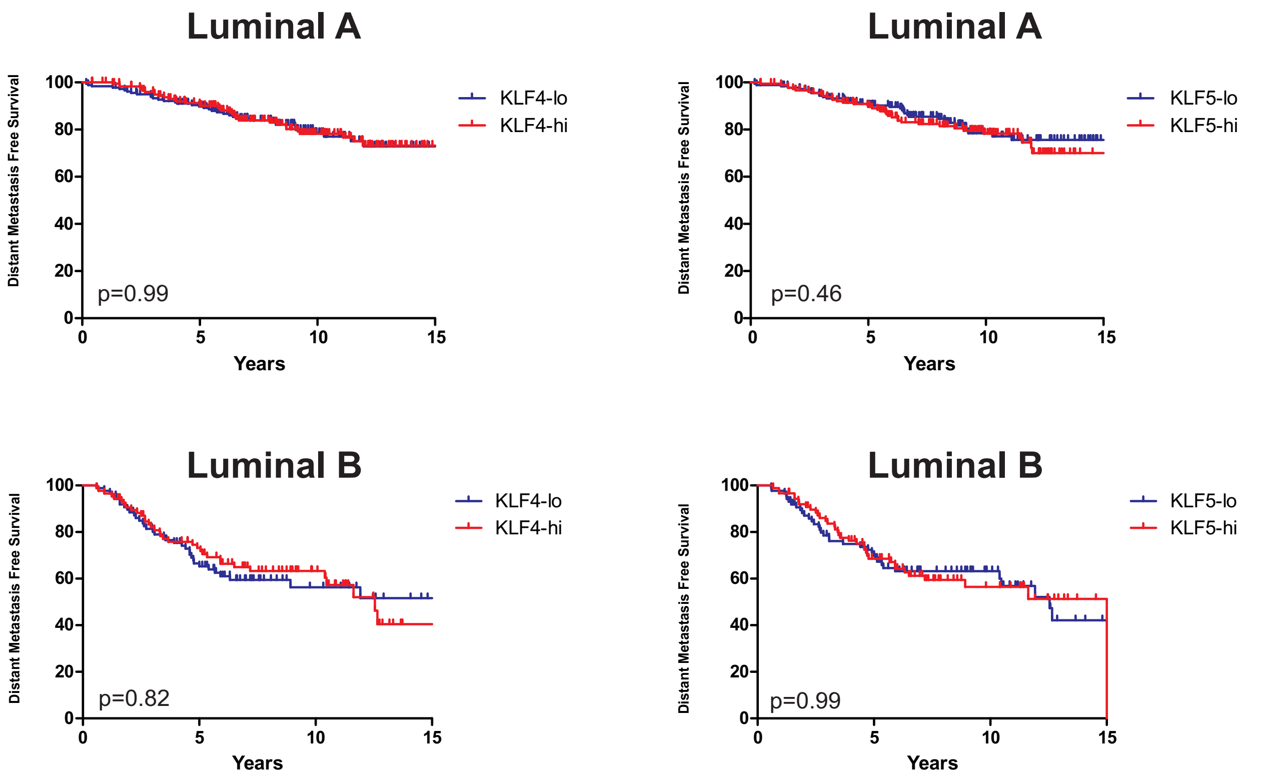

B

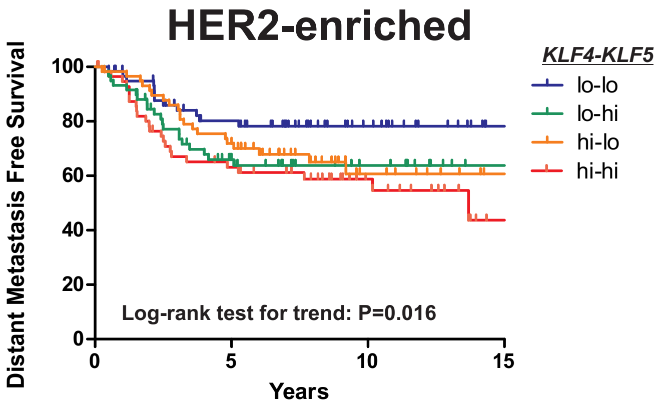

Supplemental Fig 1. DMFS analysis. A. Luminal subtype tumors were analyzed. B. The four subgroups of HER2-enriched breast cancers identified by *KLF4* and *KLF5* expression were analyzed. Patients were assigned using median expression of *KLF4* (high or low) and *KLF5* (high or low).
