# Supplementary material for: Regulation of anti-apoptotic signaling by Kruppel-like factors 4 and 5 mediates lapatinib resistance in breast cancer
Source: Cell Death Dis. 2015 Mar 19;6(3):e1699–. doi: 10.1038/cddis.2015.65 (PMC4385942; doi:10.1038/cddis.2015.65)
Supplement: Supplementary Figure 2 [file cddis201565x2.pdf]

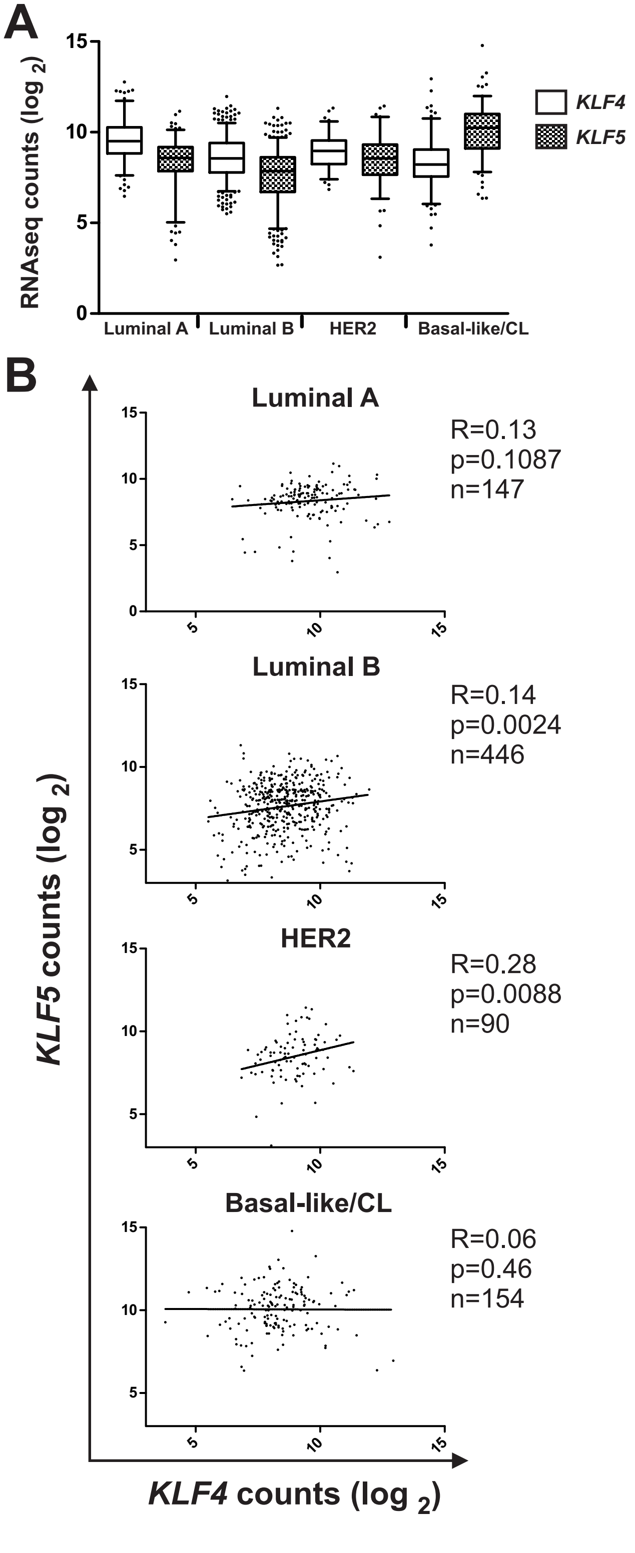

Supplemental Fig. 2. RNAseq analysis of *KLF4* and *KLF5* in primary human breast cancer samples. A) Upper quartile normalized RNAseq (Illumina HiSeq RNAseqV2) data were downloaded from TCGA and assigned a PAM50 subtype with expression displayed as a box-and-whisker plot. B) *KLF4* and *KLF5* were assessed by Spearman's correlation.
