# Supplementary material for: Regulation of anti-apoptotic signaling by Kruppel-like factors 4 and 5 mediates lapatinib resistance in breast cancer
Source: Cell Death Dis. 2015 Mar 19;6(3):e1699–. doi: 10.1038/cddis.2015.65 (PMC4385942; doi:10.1038/cddis.2015.65)
Supplement: Supplementary Table 1 [file cddis201565x3.pdf]

Primer (5'-3')	Gene
GGACCCGAGAAGACCTCCTT	musRPLP0-F
GCACATCACTCAGAATTTCAATGG	musRPLP0-R
GTGGTAAGGTTTCTCGCCTGT	MusKLF4_R_2
GGAAAAGAACAGCCACCCAC	MusKLF4_F_2
GGTCTACCACTGAGGCACTG	MusKLF5_R_2
GAGCTGGTCCAGACAAGATGT	MusKLF5_F_2
ATGCCTTTGTGGAACTATATGGC	musBCL2-F
GGTATGCACCCAGAGTGATGC	musBCL2-R
AGTGGAGGTACACCCCTCAG	musBCL-XL-F
AAGGCTGGGATCACAAACGTG	musBCL-XL-R
GACGACCTATACCGCCAGTC	musMCL1_F
AGAGGCTTCGAGTCCTTGGA	musMCL1-R
TCCCAGGTACACTTGTATGGC	HsKLF5-R
ACCCTGGTTGCACAAAAGTT	HsKLF5-F
AGAGTTCCCATCTCAAGGCA	HsKLF4-R
GTCAGTTCATCTGAGCGGG	HsKLF4-F

Supplemental Table 1: qRT-PCR primers. Listed primers were used to assay transcript levels of the respective genes. Primers are listed 5'-3'.
